# Not a general, symptom-unspecific, transdiagnostic marker for functional symptoms: sensorimotor processing of head control is intact in chronic pain

**DOI:** 10.3389/fneur.2023.1294702

**Published:** 2023-12-19

**Authors:** Franziska Regnath, Katharina Biersack, Nina Jäger, Stefan Glasauer, Nadine Lehnen

**Affiliations:** ^1^Department of Psychosomatic Medicine and Psychotherapy, University Hospital Rechts der Isar, Technical University of Munich, Munich, Germany; ^2^TUM Graduate School, Graduate Center of Medicine and Health (GC MH), Technical University of Munich, Munich, Germany; ^3^Computational Neuroscience, Institute of Medical Technology, Brandenburg University of Technology Cottbus-Senftenberg, Cottbus, Germany; ^4^Faculty of Health Sciences Brandenburg, Brandenburg University of Technology Cottbus-Senftenberg, Cottbus, Germany; ^5^Institute of Medical Technology, Brandenburg University of Technology Cottbus-Senftenberg, Cottbus, Germany

**Keywords:** somatoform, pain, functional disorder, predictive processing, perceptual dysregulation, bodily distress disorder, motor control, gaze shift

## Abstract

**Introduction:**

Functional disorders are prevalent in all medical fields and pose a tremendous public health problem, with pain being one of the most common functional symptoms. Understanding the underlying, potentially unifying mechanism in functional (pain) disorders is instrumental in facilitating timely diagnosis, stigma reduction, and adequate treatment options. Neuroscientific models of perception suggest that functional symptoms arise due to dysregulated sensorimotor processing in the central nervous system, with brain-based predictions dominating the eventual percept. Experimental evidence for this transdiagnostic mechanism has been established in various functional symptoms. The goal of the current study was to investigate whether erroneous sensorimotor processing is an underlying transdiagnostic mechanism in chronic (functional) pain.

**Method:**

A total of 13 patients with chronic (functional) pain [three patients with chronic (functional) pain disorder, F45.40, ICD-10; 10 patients with chronic pain disorder with somatic and psychological factors, F45.41, ICD-10]; and 15 healthy controls performed large combined eye-head gaze shifts toward visual targets, naturally and with increased head moment of inertia. We simultaneously measured participants' eye and head movements to assess head oscillations at the end of the gaze shift, which are an established indicator of (transdiagnostic) sensorimotor processing deficits of head control.

**Results:**

Using a Bayesian analysis protocol, we found that patients with chronic (functional) pain and control participants stabilized their heads equally well (Bayes Factor _01_ = 3.7, Bayes Factor _exclusion_ = 5.23; corresponding to substantial evidence) during all sessions of the experiment.

**Conclusion:**

Our results suggest that patients with chronic (functional) pain do not show measurable symptom-unspecific sensorimotor processing deficits. We discuss outcome parameter choice, organ system specificity, and selection of patient diagnoses as possible reasons for this result and recommend future avenues for research.

## Introduction

Chronic functional pain disorders are common, with worldwide prevalence estimates ranging to ~20% (1–4). Generally defined as pain without a structural origin (e.g., tissue damage) that could *sufficiently* explain the symptoms, functional pain is often used as an umbrella term for a range of pain phenomena, ranging from a single symptom (e.g., neck pain) to separately defined syndromes such as fibromyalgia or complex regional pain syndrome (5). In contrast to nociceptive pain (i.e., pain due to nociceptor activation) or neuropathic pain (i.e., pain due to nerve damage) affecting the structure of the body, nociplastic or functional pain—as the name already suggests—is concerned with the (dys)function of the organism: due to their symptoms, affected persons are considerably impaired in many facets of everyday functioning (e.g., physical activity, mental wellbeing, and social participation) (6), sometimes even more so compared to patients with nociceptive or neuropathic pain (7, 8). Importantly, simply relieving any (remaining) underlying structural impairment will not lead to a remission of functional pain symptoms (9). Instead, the treatment of functional pain requires a collaborative, patient-centered, multimodal approach (6, 10, 11). To date, treatment for functional pain (and functional disorders in general) is at times inadequate (12–14) and efficacy rather low (15), as the disorder commonly takes on a chronic course. Functional pain inflicts a high burden of disability, reduces quality of life, and incurs considerable individual and societal healthcare costs (12, 16).

Current neuroscientific models suggest that all symptom experience, including pain, is the result of an inferential process in the central nervous system (CNS) (17–22). At the same time, this view can also explain how pain can persist even after the initial, acute injury has fully resolved. According to Bayesian and predictive processing models of brain function, pain experience is generated from a complex interplay between “top-down” CNS-based predictions and “bottom-up” sensory input from peripheral nociceptors (23, 24). This contrasts with the Cartesian view, where pain is solely driven by nociceptive input—however, sensory processing is slow and input is often noisy, such that any response could be delayed or inadequate. To this end, the brain acts as an active agent by constantly drawing predictions about events in the body and the world, and comparing them to the actual sensory input arriving at the senses. This way, the brain only needs to process the discrepancy between the predicted and actual input—also called prediction error—thereby allowing for timely and adaptive responses. Ideally, relevant prediction errors serve to update the CNS-based internal model of the body and world, leading to more accurate predictions in the future. Importantly, top-down and bottom-up inputs are not randomly combined but weighted by their precision (or, in other words, reliability) when forming the percept. For instance, in the case of chronic (functional) pain, internal model predictions are thought to adapt during the acute illness state, as pain is a warning signal indicating actual or potential harm to the body. As structural damage slowly recovers, the brain fails to re-adapt to the healthy state, and the top-down predictions, now incorrect but still regarded as highly relevant (i.e., precise), override bottom-up input. As a result, one feels pain without an accompanying structural deficit (22, 25). Taken together, the experience of pain always arises from a combination of descending predictions and ascending input from the periphery. This also means that pain experience can be almost completely dominated by CNS-based predictions or information coming from the senses, and their relative contribution can change over time (e.g., predictions tend to dominate as acute pain becomes chronic). Importantly, the affected individual cannot discern to what extent the eventual percept was impacted by top-down or bottom-up input—the pain experience is real regardless.

Notably, this inferential process is thought to be dysregulated in functional disorders more generally (18, 20, 26–29). Functional disorders commonly co-occur, suggesting that different functional symptom presentations may share a common underlying mechanism (30–33). In fact, chronic (functional) pain may be one of the most frequent complaints co-occurring with another functional symptom (34–36). For instance, patients with functional dyspnea and healthy controls were covertly exposed to differing levels of CO_2_ as part of an experimental study using a rebreathing paradigm. Interestingly, patients reported more intense and prolonged dyspnea than objective measurements of arterial CO_2_ would suggest. In line with the predictive processing account of symptom perception, this effect was especially apparent when strong top-down predictions met relatively weak bottom-up peripheral input (37). Using the same paradigm, Van Den Houte et al. showed a similar decoupling of measured physiological responses and noted breathlessness in patients with fibromyalgia and/or chronic fatigue syndrome, where dyspnea is not a predominant symptom. Again, dysregulation in symptom perception was apparent when top-down predictions about breathlessness were strong but simultaneous bottom-up inputs were weak (i.e., normalizing CO_2_ levels), providing evidence for a general symptom-unspecific mechanism in functional disorders (38).

More experimental evidence for a shared mechanism in functional disorders comes from studies employing an eye-head paradigm, in which participants' eye and head movements are measured during large gaze shifts. Lehnen et al. compared the head stability of patients with functional dizziness and patients with structural deficits (i.e., cerebellar ataxia or complete bilateral vestibulopathy) during combined eye-head movements (39). Remarkably, all patient groups showed similar deficiencies in head motor control during both natural head movements and movements with experimentally increased head moment of inertia. The mechanism behind these initial findings was further narrowed down in a follow-up study (39), which investigated gaze stabilization in the patient group with functional dizziness: while gaze stabilization was intact during a phase where gaze was stabilized through sensory feedback mechanisms only, gaze was unstable when stabilization was dependent on correct movement planning (i.e., prediction). Together, this suggests that motor control in patients with functional dizziness may depend on excessively strong but incorrect internal model predictions, resulting in head and gaze instability due to erroneous planning of gaze shifts. To identify whether similar deficiencies in sensorimotor processing can be found more generally across functional disorders, patients with irritable bowel syndrome (IBS) performed gaze shifts in the same experimental paradigm (40). While patients' heads were stable during natural gaze shifts, patients with IBS exhibited larger head instability than healthy controls during gaze shifts with experimentally increased head moment of inertia. The results showed that patients had greater difficulties in flexibly adapting top-down predictions to the new head characteristics, pointing toward dysregulated sensorimotor processing as a symptom-unspecific, transdiagnostic mechanism in functional disorders.

Therefore, the current study examined whether erroneous sensorimotor processing is an underlying mechanism in functional pain. To this end, we measured the head stability of 13 patients with functional pain and 15 healthy controls during a gaze shift experiment, where participants performed large combined eye-head movements naturally and under an increased head moment of inertia. In line with previous studies, we hypothesized that patients exhibit larger head oscillations (i.e., instability) than controls when directing their gaze to the targets.

## Materials and methods

This project is part of the innovative training network ETUDE [(41); Encompassing Training in functional Disorders across Europe; https://etude-itn.eu/], ultimately aiming to improve the understanding of mechanisms, diagnosis, treatment, and stigmatization of functional disorders.

The current study was reviewed and approved by the ethics committee of the Technical University of Munich and was carried out in accordance with the Declaration of Helsinki. We preregistered the study on the Open Science Framework prior to any data collection. The preregistration, as well as (raw) data, analysis scripts, and files, can be openly accessed under: https://osf.io/smchp/.

All participants provided signed informed consent before any data were collected and were free to withdraw from study participation at any time. Participants received financial compensation of 10€ per hour.

### Sample

A total of 15 healthy participants (M_age_ = 46.33, SD_age_ = 15.42, nine men, six women) and 15 patients (M_age_ = 47.60, SD_age_ = 12.65, six men, nine women) with persistent chronic (functional) pain disorder [F45.40, ICD-10; World Health Organization (WHO), (42); three participants] or chronic pain disorder with somatic and psychological factors [F45.41, ICD-10; WHO (42); 12 participants] took part in the study. Patients were closely age- (BF_01_ = 2.84) and gender-matched (*X*^2^ = 0.417; BF_01_ = 1.47) to healthy controls. Healthy control participants, as well as patients, were not eligible to participate if they were under the age of 18 years, had a neurological disorder (in particular peripheral or central vestibular impairment), corrected vision of < 20% on the better eye, a hearing impairment that would not allow for experimental instruction or completion of a structural interview, acute problems of the cervical spine that would significantly prohibit the execution of head movements, or a known pregnancy. In addition, healthy controls were excluded from participation if they had a current or a history of a functional disorder or a current acute psychiatric disorder; patients were excluded if they had another functional disorder or a psychiatric disorder explaining the somatic symptoms.

Healthy controls were recruited via external and internal clinic-wide web- and poster-based announcements at the Klinikum rechts der Isar of the Technical University of Munich, Germany. Patients were recruited at the inpatient and outpatient clinics of the Department of Psychosomatic Medicine and Psychotherapy, Klinikum rechts der Isar of the Technical University of Munich, Germany.

We estimated the sample size with a power analysis based on a pilot study, which applied the same experimental paradigm to patients with functional dizziness (39). The study obtained a large significant group difference (partial η^2^ = 0.62, *f* = 1.27) in head oscillations between functional dizziness patients and healthy controls. Considering that the current study aimed to measure a transdiagnostic, symptom-unspecific mechanism, we expected a somewhat smaller group effect compared to the functional dizziness study. Assuming at least a large group effect (*f* = 0.5), the *a priori* sample size estimation using G^*^Power (43) yielded a minimum sample size of nine participants per group (α = 0.05, β = 0.8). Considering a margin of safety, we included 10 persons per group for the first round of data collection. Using a Sequential Bayes Factor design (44), we calculated a Bayesian repeated-measures ANOVA until the predetermined threshold of evidence (i.e., Bayes Factor > 3) was attained. This approach allows for flexible sampling plans and stopping rules and, thus, optimal allocation of limited resources, given our strictly defined patient sample [e.g., see (17, 45)]. We defined an *a priori* sample of 10 participants per group, which would be followed by sequential data collection of five additional participants per group at each analysis round until reaching the minimum target Bayes Factor. In the current study, one additional recruitment round was required after the initial sample, resulting in a total sample of 15 participants per group.

On the day of the experiment, all participants were clinically characterized to ensure that all *a priori*-defined study inclusion criteria were met. We conducted structured clinical interviews [SCID-5-CV, German version, (46)] to assess possible psychiatric diagnoses according to the DSM-5. We used the EyeSeeCam (EyeSeeTec GmbH, Munich, Germany) video head impulse test to examine the vestibular-ocular reflex (VOR) gain for the vertical canals by rapidly moving the participant's head in the 45-degree planes while the person maintained visual fixation on a centrally positioned LED in front of them. The ideal gain of the VOR is 1.0, meaning that head movement is optimally compensated by eye rotation in the opposite direction, thereby stabilizing gaze. We considered a deficit of the passively evoked VOR at a gain of < 0.79 (47, 48), together with re-fixation saccades, as a pathology affecting the vestibular organ located within the inner ear.

### Experimental procedure

All participants were naïve to the purpose of the experiment.

During the experiment, participants wore EyeSeeCam goggles, which simultaneously recorded head movements (recorded via 3D inertial sensors) and the left eye's movements (captured via video-oculography) in the horizontal plane in real-time at a sampling rate of 220 Hz with the EyeSeeCam measuring system (EyeSeeTec GmbH, Munich, Germany). Before starting the experimental task, we calibrated the goggles to participants' eye characteristics using horizontally and vertically aligned 5-point laser dots to ensure accurate recording.

**Figure 1:**
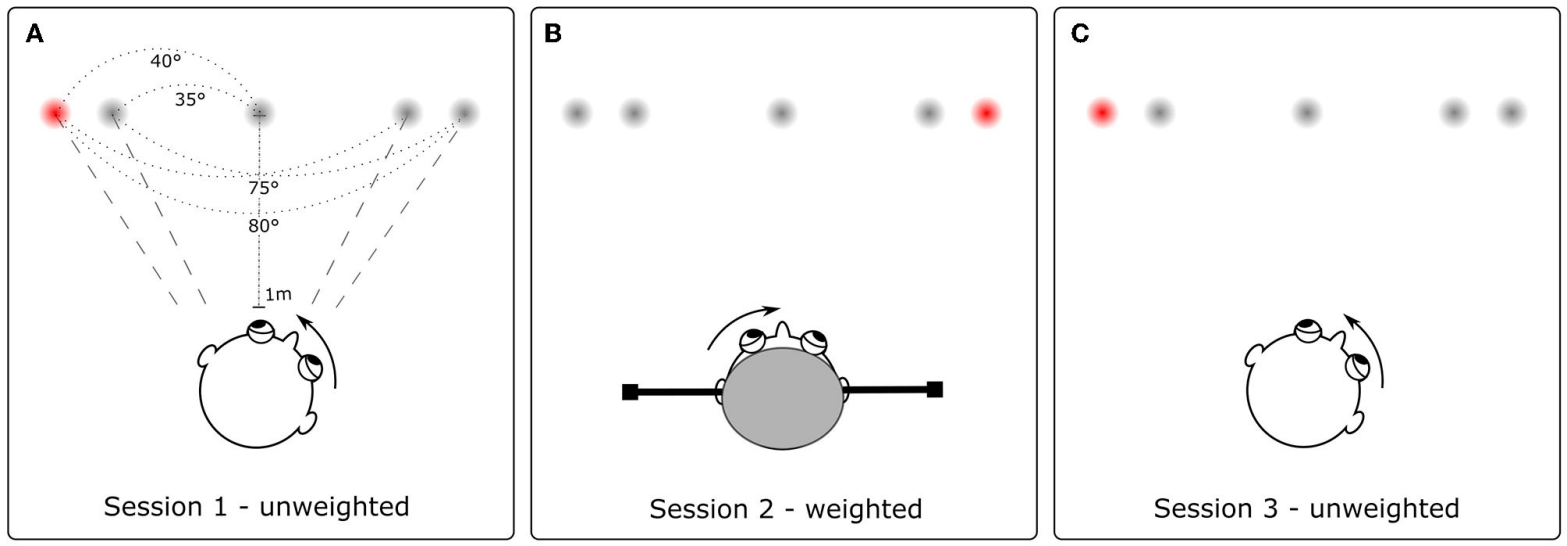
Experimental conditions. Participants performed 43 large (i.e., 75° or 80° amplitude) gaze shifts toward a red target light: first in a natural, unweighted condition **(A)**, followed by a weighted condition **(B)** with an increased head moment of inertia, and finally again in an unweighted condition **(C)**. In the weighted condition **(B)**, participants were required to flexibly adapt to the new head characteristics to optimize head stability during gaze shifts. Including two unweighted conditions at the beginning **(A)** and end **(C)** of the experiment allowed us to explore potential learning effects over time.

In total, participants successively completed three experimental rounds (Figure 1): first, an unweighted condition, while only wearing the measurement goggles; second, a weighted condition, where participants were wearing the measurement googles as well as a helmet with eccentrically placed masses that increased the head moment of inertia by 3.1-fold; finally, again an unweighted condition identical to the first round.

While seated in front of five red LED lights,^1^ the participants' task was to make combined eye-head gaze shifts toward a briefly flashing (one at a time) LED target and to keep their gaze on the target until the next light flashed. We explicitly instructed participants to use both eyes and head when directing their gaze, as they would when naturally looking around the room. In addition, we asked subjects to only shift their gaze when the next target flashed and to refrain from already moving in anticipation in case they had detected a pattern in the flashing of the LED lights. When participants completed the second weighted experimental round, we hid the helmet from the participant's view until the experiment was completed. The experimenter introduced the helmet as a bike helmet and gave no further information on its characteristics (e.g., weight, shape, and modifications). In addition, we instructed participants not to move their heads until the experimental session started again. That way, participants were not able to adapt to the new head characteristics before performing the first gaze shift while wearing the helmet.

**Figure 2:**
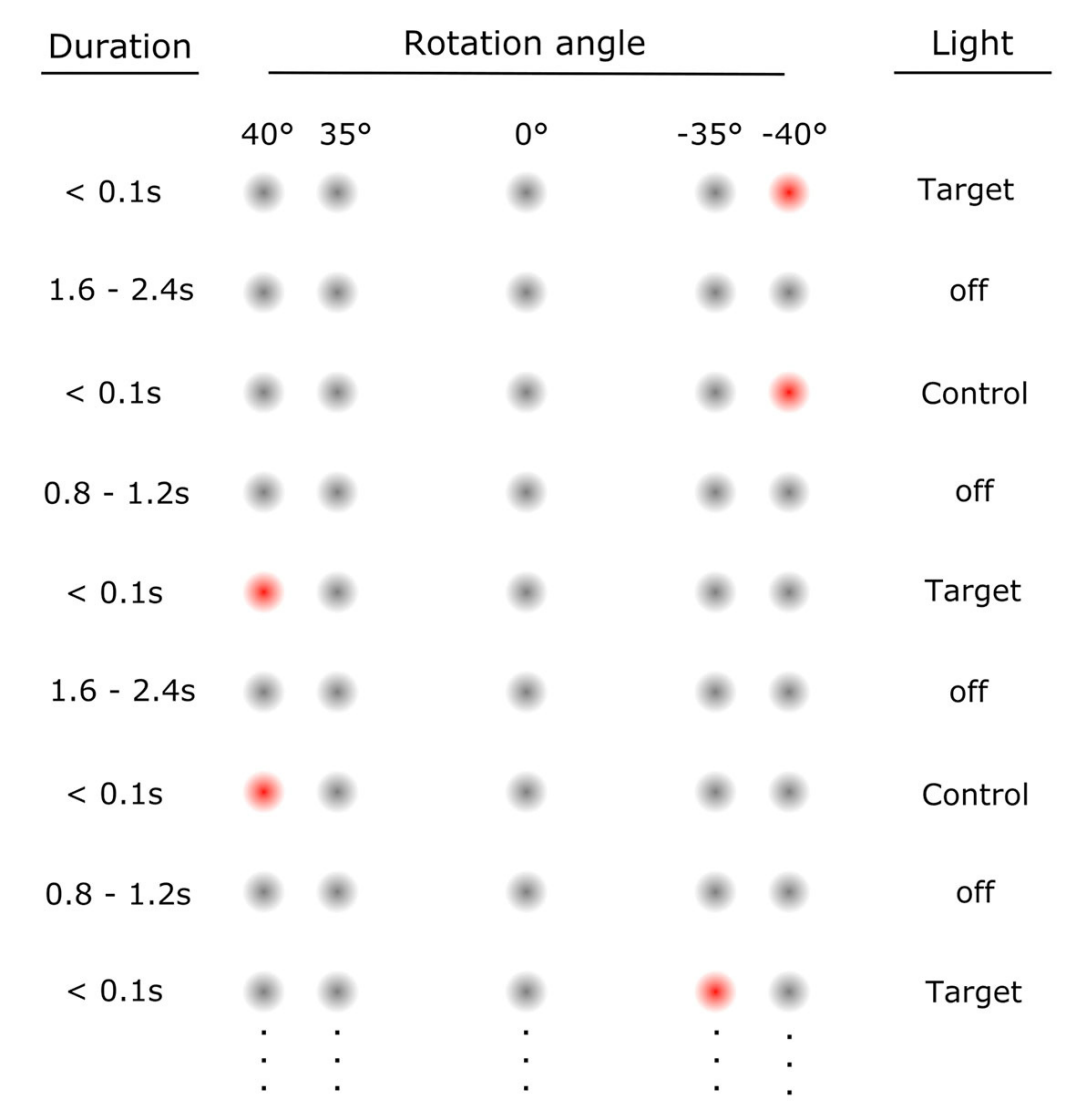
Illustration of LED sequence in the experiment. Each target light (flash <0.1 s) was followed by a control light, which briefly flashed (<0.1 s) in the same location to allow participants to correct their gaze if needed. Target light and control light were separated by an inter-trial interval (ITI, 1.6–2.4 s lights off). This ensured that subjects were more likely to start out at the intended position before the next target flashed. The control light was followed by an ITI (0.8–1.2 s lights off), after which the next target lit up. Overall, ITIs were randomized to avoid anticipatory movements toward the next predicted target. We express the amplitude of the required rotation to each light in degrees. With the center LED corresponding to 0°, negative angles represent clockwise rotations and positive angles rotations in the counterclockwise direction in the horizontal plane. Thus, targets lit up in the following sequence: 0° 0° −40° 40° −40° 40° −35° 40° −40° 40° −35° 40° −40° 35° 0° −40° 40° −40° 35° −40° 40° −40° 35° −40° 40° −35° 40° −35° 40° −40° 35° −40° 35° −35° 40° −35° 35° −40° 40° −35° 35° −40° 40° −35° 35° −40° 40° −40° 40° −35° 40° −40° 0°. Control lights are not specially indicated here but always lit up after the target light in the same position. Note that the first central light at 0° was continuously illuminated for 10 s, as it served as a reference for the initial head position for later data processing.

Participants completed the task in complete darkness; target LED lights were only flashed briefly (<0.1 s), so participants' executed gaze shifts were not reliant on visual input. In total, participants carried out 52 gaze shifts, of which 43 were large (i.e., 75° or 80°) gaze shifts. Figure 2 demonstrates the LED sequence and illustrates an example.

### Eye-head paradigm: feedforward and feedback

Measuring head oscillations as an outcome of sensorimotor processing during active head movements, such as those performed in this eye-head experiment, allowed us to examine the interaction of internal model predictions and processing of sensory signals from the peripheral body.

When visual^2^ (e.g., receptors located in the eye), proprioceptive (e.g., neurons in the neck muscles), and vestibular (i.e., semicircular canals and otoliths located in the inner ear) systems sense a movement (e.g., rotation and acceleration of the head), this sensory information is sent to the brain for further processing. For passive head movements (e.g., when driving on a bumpy road), movements of the head are unexpected and, therefore, no predictions about the sensory consequences can be formed *a priori*—any perturbations to the head must be processed in the brain, and resolved *a posteriori* based on sensory input. However, for active head movements, the brain also generates *a priori* predictions of how an active movement will impact the sensory organs (i.e., a reafference estimate based on the efference copy of the motor command). This way, when the planned, actual movement is executed, only information that deviates from this prediction—the mismatch between reafference and reafference estimate (i.e., the prediction error)—needs to be processed further in the brain and can be used as feedback about the executed movement. This allows for efficient (i.e., only prediction errors are processed), timely (i.e., prepared motion rather than stimulus-response), and more accurate (e.g., taking into account past experiences and context) movements [e.g., see (49–53)].

For instance, active head motor commands based on an inaccurate internal model of the head biomechanics will result in a suboptimal head movement (e.g., head oscillation), inducing prediction errors that are ideally used as feedback to update the central nervous system-based internal representation of the head. With the eye-head paradigm employed in the current study, we are able to experimentally induce a mismatch between the internal model of the head plant and the new head characteristics by placing a weighted helmet on the participant's head: the increased head moment of inertia (3.1-fold) is not yet represented in the internal model, leading to measurably increased head oscillations. The resulting prediction errors should then serve to update the model to the new head properties, resulting in more accurate head movements over the course of these trials (39, 54–57).

## Data analysis

### Data (pre-)processing

We used MATLAB (58) to preprocess raw data, and Python programming language [(59); Python Software Foundation, https://www.python.org/] to manually inspect and, if required, correct automatically detected events in the data and prepare data for subsequent analysis. Finally, we used the statistical software program JASP (60) to conduct statistical tests. All analysis scripts can be accessed at: https://osf.io/smchp/.

**Figure 3:**
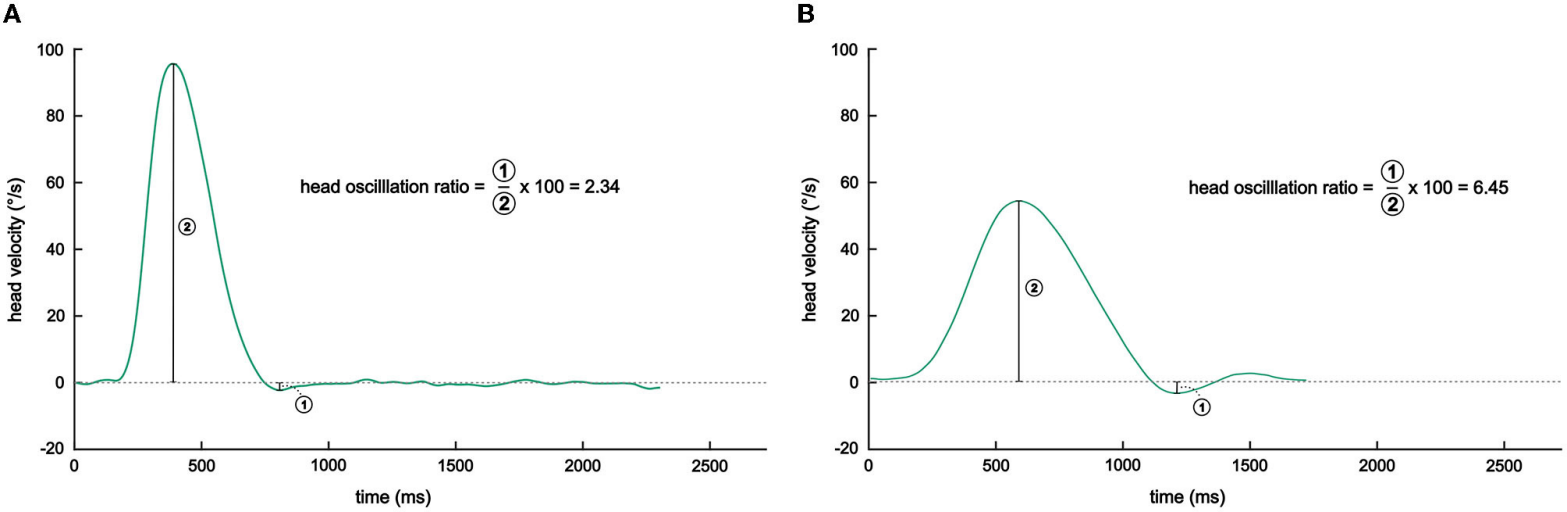
Illustration of head oscillation ratio calculation. Shown are representative raw head velocity traces of a patient during a gaze shift in the **(A)** unweighted (session 1) and **(B)** weighted sessions. Note how the head oscillation (i.e., the undershoot in relation to the peak head velocity) is markedly increased when the participant performed the gaze shift while wearing the helmet, reflecting a mismatch in the planned and actual head movement.

During data preprocessing, we applied a 20-Hz Gaussian low pass filter to continuous, raw eye pupil and raw head position (in deg) and velocity (in deg/s) data streams. Head velocity data were directly obtained from recordings of the 3D inertial sensors, head position was derived from numerical temporal integration of head velocity, and eye position in the horizontal plane was computed from pupil rotation recordings. We then separated the continuous data streams into 52 trials, such that one trial corresponds to one horizontal eye-head gaze shift toward the target light: target LED onset denoted the start of the trial, and control light onset denoted the end of the trial period. For subsequent analysis, we only considered gaze shifts with a target gaze amplitude of 75° or 80°, which resulted in 43 trials per session (i.e., 1—unweighted, 2—weighted, and 3—unweighted) per participant. We *a priori* determined the head oscillation ratio as the primary outcome variable of this study, defined as the absolute ratio of the first positive head velocity peak and the first subsequent negative peak of head velocity [in analogy to (39, 61)]—this way, oscillations are normalized by peak velocity in a given trial (see Figure 3). To this end, head peak velocity, the subsequent first zero crossing (i.e., where head velocity first reaches zero and becomes negative), the absolute minimum peak of the first undershoot (where the head momentarily comes to a halt and then moves backward), and the second zero crossing (i.e., head velocity reaches zero again and then becomes positive) were initially automatically detected and the head oscillation ratio of each trial computed.

Next, we manually inspected each trial and corrected any automatic detection errors. Automatic detection usually failed when participants moved their gaze too early (i.e., in anticipation of the next target flash) or initialized movement too late. Therefore, if participants shifted their gaze too early, we extended the trial window to also include the period between the prior control light and target onset. Similarly, if participants shifted their gaze too late, we extended the window to also consider the movement after the control light had already flashed. We excluded the trial if peak head velocity and/or undershoot could (still) not be determined with certainty. This way, we were able to minimize data loss and include as many gaze shifts as possible in our analyses. On average, patients moved too early in 1.3% (SD = 2.4%) of trials and too late in 4.4% (SD = 4.6%) of trials, whereas 1.5% (SD = 2.5%) of trials had to be excluded because we could not determine all parameters necessary to compute the head oscillation ratio. Healthy control participants, on average, moved too early in 0.3% (SD = 0.5%) of trials and too late in 5.1% (SD = 5.4%) of trials, and 1.7% (SD = 2.5%) of trials had to be excluded from further analysis.

Following, we excluded all trials with a gaze amplitude of <40°, computed as the sum of head position and eye position in the horizontal plane. For trials with gaze shifts that were executed too early or too late, we also considered the extended movement window when calculating gaze amplitude. Overall, we excluded (on average) 6.7% (SD = 6.0%) of trials per patient and 4.7% (SD = 2.0%) of trials per control participant because gaze shifts were too small. Furthermore, within each participant's session, we excluded trials with a head oscillation ratio that was more than two standard deviations below or above the average head oscillation ratio. Consequently, we removed, on average, 4.1% (SD = 1.1%) and 4.9% (SD = 1.0%) of trials per patient and healthy control participant, respectively. Overall, this resulted in, on average, 38.54 trials (SD = 2.03) in the first session (unweighted 1), 38.08 trials (SD = 3.2) in the second session (weighted), and 39.00 trials (SD = 2.16) in the third session (unweighted 2) to be included for patients; for participants in the control group, we could include 38.77 trials (SD = 1.64) for the first session (unweighted 1), 38.92 trials (SD = 1.8) for the second session (weighted), and 39.08 trials (SD = 1.38) for the third session (unweighted 2), on average. Note that the reported percentages of anticipated/delayed gaze shifts and trial exclusions (oscillation ratio undetermined, gaze amplitude <40°, outliers) pertain to all included participants in the final analysis round.

Finally, we re-calculated the mean head oscillation ratio with the remaining trials per session (i.e., unweighted 1—weighted—unweighted 2) and per participant to be entered for subsequent statistical analysis.

### Statistical analysis

We employed a mixed design, where each participant completed all three sessions successively but belonged to one of two groups. Therefore, for statistical analyses, we performed a Bayesian repeated-measures (RM) ANOVA, where the variable session was the within-subjects factor (three levels: 1—unweighted, 2—weighted, and 3—unweighted) and group (two levels: patient and healthy control) was the between-subjects factor, with the oscillation ratio as the dependent variable.

Unlike the more traditional null hypothesis significance testing (NHST) approach, the employed Bayesian statistical approach allowed us to examine the relative evidence for both the null (i.e., absence of a group and/or session effect) and alternative hypothesis (i.e., presence of a group and/or session effect). More specifically, the Bayes Factor (BF) quantifies the graded strength of statistical evidence for a specific model or effect, where BF subscripts—BF_01_ or BF_10_–indicate support for the null hypothesis and the alternative hypothesis, respectively. We use quantitative labels as described in Wagenmakers et al. (62) to interpret the evidential strength: BFs of 1, 1–3, 3–10, 10–30, 30–100, and >100 classify no, anecdotal, substantial, strong, very strong, and extreme evidence, respectively. For instance, a BF_01_ of 4 would denote that the data are four times as likely to have occurred under the null than under the alternative hypothesis, thus representing substantial support for the null hypothesis vs. the alternative hypothesis. For model comparisons, we report the BF for the model of interest compared to a null model only including the subject; for analysis of effects, we report the BF_inclusion_ (or BF_exclusion_) that reflects the evidential strength of all models containing a particular effect compared to all models without the effect. This analysis plan was preregistered and can be accessed at: https://osf.io/me4zc/.

## Results

For the first interim analysis (n_patient_ = 10, n_control_ = 10), two patients had to be excluded from the analysis because they did not follow the experimenter's instructions: one patient only performed gaze shifts in anticipation of the target light, while another patient already moved the eyes to the expected position of the next target before also moving the head once the target finally flashed. Thus, we calculated a Bayesian RM ANOVA with the remaining eight patients and 10 healthy control participants, which revealed anecdotal (BF_01_ = 2.80, BF_exclusion_ = 2.63) evidence for no differences in the head oscillation ratio between patients and controls across all three sessions. Analyses revealed that our experimental manipulation of increasing the head moment of inertia (i.e., a mismatch between predicted and actual sensory feedback) was successful, as all participants exhibited larger head oscillations during the weighted session (BF_10_ = 2.211e+11, BF_inclusion_ = 1.706e+11, extreme evidence) than during both unweighted sessions. In addition, *post-hoc* analyses revealed a learning effect from the first unweighted to the second unweighted session: irrespective of group membership (BF_01,U_ = 3.27), participants were able to reduce head oscillations over the course of the experiment (BF_10,U_ = 31.74).

**Figure 4:**
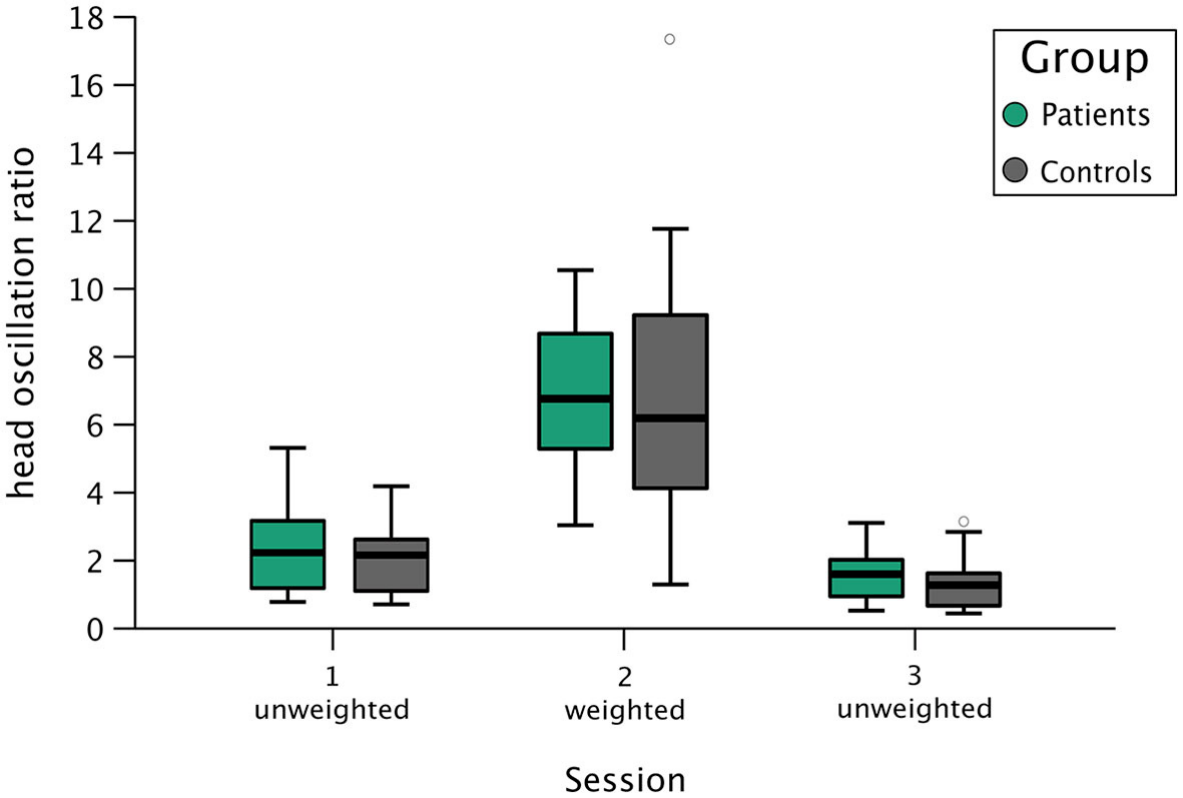
Group results of head oscillation ratios during all three experimental sessions. This box plot shows group results for patients (boxes in green) and healthy controls (boxes in gray) during session 1 (unweighted), session 2 (weighted—with helmet), and session 3 (unweighted). The y-axis depicts the head oscillation ratio, where a larger value corresponds to larger head oscillations at the end of the combined eye-head gaze shift. Each box represents the interquartile range (IQR), delineating the lower (25th percentile) and upper (75th percentile) quartile, with the vertical line depicting the group's median head oscillation ratio during the respective session. The boxes' whiskers correspond to the most extreme data point within 1.5 times the IQR from the lower and upper quartiles. Observations exceeding this range are marked as a gray circle, representing a healthy control participant in the weighted condition and a different control participant in the second unweighted condition.

For the second analysis round, we recruited five additional participants per group and performed statistical analyses in line with round 1. With a sample of 15 healthy controls and 13 patients (two patients were excluded during round 1), the Bayesian RM ANOVA yielded substantial (BF_01_ = 3.7, BF_exclusion_ = 5.23) evidence for the absence of a group difference in head oscillations (Figure 4). Again, the experimental manipulation of experimentally inducing a mismatch between old and new head characteristics by increasing participants' head moment of inertia was successful: we obtained extreme (BF_10_ = 2.684e+15, BF_inclusion_ = 2.729e+14) evidence that participants exhibited increased head oscillations when wearing the helmet. Matching the analysis in round one, *post-hoc* analyses also indicated extreme (BF_10,U_ = 3,215.99) evidence for a learning effect: participants reduced head oscillations from the first to the second unweighted session, regardless of group affiliation (BF_01,U_ = 4.38).

Overall, the analyses of round two were in line with the results obtained in round one (i.e., in terms of direction of effects) but were larger in the magnitude of evidential strength. Since we crossed the *a priori*-defined mark of substantial evidence (BF_01/10_ > 3), we terminated data collection with this round. Table 1 provides a brief overview of head oscillation values within sessions and groups in this final round.

**Table 1:** Descriptive statistics of head oscillation ratios for patients (*n* = 13) and healthy controls (*n* = 15) during each experimental session.

	**Session 1 (unweighted)**		**Session 2 (weighted)**		**Session 3 (unweighted)**	
	**Patients**	**Controls**	**Patients**	**Controls**	**Patients**	**Controls**
Mean	2.35	2.12	6.79	7.04	1.53	1.42
SD	1.39	1.20	2.27	4.29	0.76	0.88

Finally, of all 13 included patients in the final analysis round, 61.5% reported pain in the leg/knee/foot region, 46.2% in their spine, 38.5% in the head/face area, 30.8% in the neck, 30.8% in the arms/hands, 15.4% in the shoulder, 7.7% in the abdomen, 7.7% in the genital area, and 7.7% in the pelvis. Note that the majority of patients experienced pain in more than one body area. Since patients were recruited from in- and outpatient units for severe functional disorders, they were suffering from chronic (functional) pain that significantly interfered with and limited their personal, social, and occupational lives. That is, 30.8% reported being unable to work, while 38.5% were still employed but experienced substantial impairment at work, and 69.2% felt that they could not fully partake in their everyday personal (e.g., hobbies, grocery shopping, and clothing themselves) and social lives (e.g., meeting friends) due to their symptoms. Table 2 summarizes the onset, frequency, and characteristics of pain symptoms described by patients, and Table 3 provides an overview of the medication taken at the time of measurement by patients and healthy controls.

**Table 2:** Descriptive summary of onset, frequency, duration, and characteristics of pain symptoms as reported by patients (*n* = 13).

**Patient**	**Onset (in years)**	**Frequency (days per week)**	**Duration (in hours)**	**Characteristics**
P1	4	/^a^	/^a^	Stinging, pressing
P2	9	7	variable^b^	Stinging, cold
P3	19	7	16–24	Stinging, prickly, rubbing
P4	1	7	16–24	Burning, itching, pulling
P5	7	Not daily^b^	21	Stinging
P6	7	2–3	2–3	Throbbing, shooting, burning
P7	30	2–3	0.1–24	Dull, pressing, stinging
P8	3	7	16–24	Shooting, stinging, burning
P9	25	3–4	16–24	Stinging, pressing
P10	6	7	16–24	Dull, pressing, throbbing
P11	14	7	24	Dull, stinging, pulling, throbbing
P12	3	7	16–24	Stinging, sore, pulling
P13	8	7	24	Burning, stinging

a Information was not collected at measurement.

b Participant could not give a definitive answer, or symptoms were too variable to report a pattern.

**Table 3:** Overview of medication (active ingredient) intake of participants in the patient (*n* = 13) and control (*n* = 15) group.

**Subject**	**Medication (dose, intake frequency)**
P1	Denosumab (60 mg, every 6 months)
P2	Pregabalin (75 mg, 1x day), Duloxetine (60 mg, 1x day)
P3	Novaminsulfon (500 mg, 3x day; double if necessary), Venlafaxine (225 mg, 1x day), Metformin (500 mg, 2x day), Atorvastatin (40 mg, 1x day), Pramipexol (0.35 mg, 1x day), Macrogol (13.125 mg, 2x day)
P4	Lacosamid (100 mg, 2x day), Lamotrigin (25 mg, 1x day)
P5	Statin (no information on dosis, 1x day)
P6	Penicillin (1.5 Mega IE, 2x day), Tapendadol (100 mg, 2x day)
P7	Atorvastatin (20 mg, 1x day), Ibuprofen (if necessary), CBD-oil (if necessary), sumatriptan (if necessary)
P8	Tilidine (50 mg, 1x day), Pregabalin (5x 100 mg, 2x day)
P9	Salmeterol xinafoate and Fluticason 17-proprionate (if necessary), Ibuprofen (if necessary)
P10	Acetylsalicylic acid (250 mg, if neccessary), Paracematol (250 mg, if necessary), Amitriptyline (12.5 mg, 1x day)
P11	Gabapentin (500 mg, 3x day), Pantoprazole (20 mg, 1x day), Magnesiumoxide, heavy (250 mg, 1x day), Celecobix (100 mg, if necessary), Ibuprofen (800 mg, if neccessary)
P12	Amitriptylin (6x 45.3 mg, 1x day)
P13	Pregabalin (330 mg, 2x day), Duloxetine (33.7 mg, 1x day), Acetylsalicylic acid (100 mg, 1x day), Rosuvastatin (20.8 mg, 1x day), Pantoprazole (20 mg, 1x day), Tamsulosin (0.4 mg, 1x day)
C5	Exemestan (25 mg, 1x day)
C6	Olmesartan (20 mg, 1x day), Amlodipine (5 mg, 1x day)
C7	Levothyroxine (no information on dosage)
C11	Levothyroxine (88 μg, 1x day)
C12	Valsartan (no information on dosage), Salbutamol (spray, 2x day)
C14	Levothyroxine (no information on dosage)

P1–P13 denotes patients, while C5–14 denotes healthy controls. In this table, we only listed healthy controls who reported medication intake; the remaining nine healthy control participants (i.e., C1–4, C8–10, C13, C15) took no medication at the time of measurement.

## Discussion

The goal of this study was to experimentally examine whether erroneous sensorimotor processing is a transdiagnostic mechanism underlying chronic (functional) pain. To this end, we measured head stability in a group of 13 patients with functional pain and 15 healthy controls when they performed large gaze shifts naturally, with experimentally increased head moment of inertia, and again naturally. Contrary to our hypothesis, we found that head stability during all three sessions did not differ between patients and healthy control participants. This suggests that sensorimotor processing was intact in our chronic (functional) pain patient group.

In addition, we found that both patients and healthy controls were able to reduce head oscillations from the first unweighted (pre-weight) to the second unweighted (post-weight) session. Presumably, the weight of the measurement goggles had already induced a slight increase in the head moment of inertia, leading to a mismatch in predicted and actual head movement characteristics. As a result, head oscillations were slightly increased toward the beginning of the experiment, and both groups were equally able to adjust their internal model predictions to the altered head characteristics throughout the experiment. Possibly, the even stronger prediction errors evoked during the weighted condition were (additionally) driving this CNS-based learning process. A similar learning effect was already observed in an earlier study with patients suffering from IBS (*n* = 7), which employed the same paradigm (40).

### Altered sensorimotor processing in pain

In light of the predictive processing model, pain experience is not solely the product of nociceptor activation but rather represents the final product of a complex interplay of CNS-based internal model predictions and peripheral input. Any mismatch between the expected and actual sensory feedback produces a prediction error, which should prompt an update of the internal model. However, the brain may categorize an underlying inconsistency as potential bodily damage or injury and consequently generate the perception of pain as a protective warning signal. Pain may be a consequence of erroneous sensorimotor processing, but at the same time, it is also a cause: an individual suffering from chronic pain may restrict interaction with the environment (e.g., because of mental exhaustion and stiffness) and may be required to adapt existing motor control patterns (e.g., compensate for a painful leg). In turn, this can lead to additional strain and pain (e.g., due to avoidance of movement) or movement that constantly produces prediction errors (e.g., due to irregularities in new movement strategies). In fact, a broad range of functional pain conditions are closely linked to altered sensorimotor processing and, more specifically, adaptations in motor control and planning [(63–65), for a comprehensive review, see (66)]. This view is corroborated by neuroscientific evidence suggesting that dysregulated sensorimotor processing in functional pain is associated with altered neural representation in the brain [see (67) for an overview, (68–70)]. For instance, adaptions in the primary somatosensory cortex after exercise therapy have been linked to reductions in pain intensity in CRPS (71). Therefore, we expected to be able to measure such processing deficits in our movement-based experiment, where sensorimotor processing was specifically challenged.

### Head instability as a transdiagnostic marker of erroneous sensorimotor processing in functional disorders

Prior research employing this paradigm has observed increased head oscillations already during natural, unweighted gaze shifts (i.e., while only wearing the measurement goggles) in patients with functional dizziness (39), indicating that head properties were not represented accurately in the central nervous system-based internal model and were also not sufficiently updated despite prediction errors resulting from the suboptimal, oscillating head movement. Notably, the patients' experienced symptoms could—at least in part—be an expression of similar head instability in everyday life since head oscillations fit well with the reported dizziness symptomatology. Similarly, Schröder et al. (40) measured increased head oscillations in patients with irritable bowel syndrome (IBS; *n* = 7) and observed increased head oscillations only during gaze shifts with increased head moment of inertia, suggesting that resulting prediction errors did not sufficiently update the internal model at the same rate as it did in healthy controls. The lack of increased head oscillations in both natural sessions fits well with the fact that patients with IBS did not experience any dizziness symptoms. However, the observed pronounced head oscillations in the weighted session point toward a general, transdiagnostic sensorimotor processing deficit in this patient population. Thus, IBS may be understood as a sensorimotor processing disorder, where motor control is not only impaired symptom-specifically (e.g., gut motility) but perhaps also more generally throughout the body (e.g., head control). This is also more in line with the structural makeup of the brain, which is not divided into entirely separate motor regions for each body part (e.g., gut vs. head) but rather coordinates movement throughout the whole body within a complex motor network (72).

In sum, we suggest that these earlier findings may point toward a transdiagnostic, symptom-unspecific sensorimotor processing deficit in functional disorders more generally, which can be unraveled when challenged experimentally within the presented eye-head paradigm: by increasing the head moment of inertia (3.1-fold) with our helmet, we are able to introduce a very specific perturbation to the sensorimotor system that produces a definite prediction error and erroneous movement (i.e., head oscillation), which can be compared to an optimal response (i.e., smooth head movement). Importantly, the observed difficulty in dealing with perturbation could be a general problem in functional disorders and possibly represent a vulnerability for developing additional functional symptoms in the future (e.g., dizziness)—a phenomenon that is also commonly seen in clinical practice [e.g., (73, 74)]. Therefore, to extend this line of research, the current study examined whether a similar symptom-independent sensorimotor processing deficit may also be present in chronic (functional) pain. Although the presented negative results refute the presence of a similar transdiagnostic marker in this patient group, they are an important contribution to the current body of evidence, as negative or null results often remain unpublished, leading to a bias in the available literature (75, 76).

### Erroneous sensorimotor processing: not a measurable marker in chronic (functional) pain

In the following section, we outline three possible reasons for the obtained negative result of this study.

First, head stability as a marker of sensorimotor processing deficits may not be the appropriate parameter to measure a transdiagnostic mechanism in functional pain. For example, some experimental paradigms directly measure altered pain perception in functional pain syndromes [e.g., thermal grill illusion; (77)]. However, to reveal a potentially unifying, transdiagnostic mechanisms in functional disorders generally and functional pain specifically, experimental setups that examine performance in symptom-unrelated modalities are necessary. For instance, Cost et al. (78) showed that patients with fibromyalgia exhibited significant disturbances in balance and gait compared to healthy control participants. However, these objective measurements also matched patients' subjective reports on motor impairment in everyday life and thus did not provide insight into kinematic parameters as a transdiagnostic marker in functional (pain) disorders [see also (79)]. To the best of our knowledge, so far, only two studies (38, 40) have provided direct experimental evidence of a symptom-unspecific, objectively measurable transdiagnostic marker of erroneous sensorimotor processing in functional (pain) disorders and have been discussed earlier in more detail.

Second, pain symptomatology may simply be too far removed from the organ systems involving vestibular processing and (eye-head) motor control to measure any underlying transdiagnostic mechanism.^3^ Although plausible, this still raises the question of why we were able to measure processing deficits in IBS (*n* = 7) (40) with the same paradigm, where the affected organ systems seem similarly far removed from those challenged in the experiment. Furthermore, diagnostic criteria of IBS include pain (associated with defecation or stool frequency/appearance) as a hallmark symptom (42, 80, 81). Similar to functional pain, pain in the context of IBS also seems to be, at least in part, a result of dysregulated processing in the CNS (80, 82–85). However, it is possible that the measured transdiagnostic sensorimotor processing deficits observed in the IBS sample are independent of the pain symptomatology and are instead a marker of other gastrointestinal symptoms in IBS, such as intestinal motor abnormalities and excessive contractile activity (86–89). Unlike patients with IBS, pain in the current sample was not focused on abdominal pain only but could differ widely in site and spread. In sum, patients with functional pain may not exhibit an all-encompassing deficit in adapting internal models but may instead experience dysregulated adaption more restricted to symptom-specific internal models (e.g., those concerned with potential damage in the body).

Third, patients were eligible to participate if they had been diagnosed with either persistent chronic (functional) pain disorder [F45.40, ICD-10, (42)] or chronic pain disorder with somatic and psychological factors [F45.41, ICD-10, (42)]. Notably, F45.41 was the predominant diagnosis, with 10 out of 13 included patients in our analysis. Although both labels outline the diagnosis as persistent pain not *sufficiently* explained by an underlying structural impairment, a diagnosis of F45.40 posits that psychosocial stress factors play a major role in the onset, severity, exacerbation, or maintenance of pain symptoms, while F45.41 requires structural processes (e.g., damaged tissue) to cause the initial pain complaint, with psychosocial factors subsequently contributing to pain intensity, exacerbation, or maintenance (90). In other words, patients with an F45.41 diagnosis can suffer from pain that is a result of structural impairment *as well as* centrally mediated (functional) processes *simultaneously*. That is, most patients in the current sample may suffer from pain that stems, to a larger part, from an underlying structural impairment and, perhaps to a lesser extent, from an underlying functional CNS-processing impairment. In contrast, earlier studies using the same paradigm found measurable head instability in dizziness (*n* = 8) (61) and IBS (40) patients (*n* = 7), where extensive neurological or gastrointestinal workups did not reveal any comorbid organic-structural impairment. Taken together, our experimental paradigm may not have been sensitive enough to capture possible minor, transdiagnostic, sensorimotor processing deficits in the current functional pain sample.

## Limitations

The findings of the present study should be interpreted in light of the following limitations. First, our sample size is relatively small, which may limit the generalization of the obtained effects. The reason for the small number of participants is the nature of our patient sample: only patients with an isolated functional pain disorder were eligible to participate since our goal was to measure a possible transdiagnostic marker. This severely limited the selection of patients, as comorbidity among functional symptoms is the rule rather than the exception (73, 74, 91). Moreover, it is important to note that a similarly small sample size was able to detect such sensorimotor deficits in patients with IBS [(40); seven patients]. In addition, we recruited patients from a specialized institution for functional disorders, which makes it likely that patients suffered from more severe forms of functional pain and additional psychiatric disorders than is typical for this population. Finally, because we selected patients only based on diagnostic labels (i.e., F45.40 or F45.41), the affected region (e.g., head vs. foot) and spread (e.g., isolated body part vs. whole body) of pain differed considerably among participants. However, we did not expect these factors to meaningfully impact sensorimotor processing in the context of our experiment, especially because we aimed to measure sensorimotor processing deficits as a more general, transdiagnostic mechanism.

## Future research and hypotheses

The current study adds a valuable contribution to the current body of evidence, as experimental studies on transdiagnostic mechanisms in functional disorders are generally scarce (41). Future research could focus on re-evaluating head stability in the context of the employed eye-head paradigm as a marker to measure transdiagnostic mechanisms in functional (pain) disorders. Calculating the oscillation ratio based on head velocity traces has proven to be an adequate marker of erroneous sensorimotor processing (39, 40, 54–56), but different head velocity parameters (e.g., area under curve and skewness) may be worth validating in subsequent studies. Furthermore, in line with earlier studies on optimal control of head movements in the eye-head paradigm (92), computational modeling could be applied to further narrow down the interplay of CNS-based prediction and sensory input in functional pain. In addition, we recommend analyzing the gaze stability^4^ of patients with functional pain during natural and weighted gaze shifts, akin to the previous study on patients with functional dizziness (61). For instance, it may be possible that eye movements do not sufficiently counteract the ongoing head movement [i.e., suboptimal motor planning (92)] and thus still reveal sensorimotor processing deficits that cannot be seen in head stability alone.

When examining possible transdiagnostic mechanisms underlying functional (pain) disorders, future studies could also employ different experimental paradigms (e.g., with a measurement modality slightly closer to the modality of pain) or the same eye-head paradigm with a different patient sample. That is, the eye-head paradigm might reveal processing deficits if patients suffering from other functional pain syndromes are measured. For instance, fibromyalgia may be a suitable choice, as earlier studies have already shown transdiagnostic markers in patients with this diagnosis [i.e., dysregulated breathing perception (38)]. Alternatively, it would be interesting to test patients with other functional disorders (e.g., functional movement disorder) with the eye-head paradigm, where the affected organ system is tied closer to the vestibular system or (eye-head) motor control in general. Complex regional pain syndrome might be another candidate for testing, as pain regions are clearly defined and limited, and top-down pain-regulating mechanisms are known to play an important role (93).

## Data availability statement

Raw data supporting the conclusions of this article are available under https://osf.io/smchp/.

## Ethics statement

The studies involving humans were approved by Ethics Committee of the Technical University of Munich. The studies were conducted in accordance with the local legislation and institutional requirements. The participants provided their written informed consent to participate in this study.

## Author contributions

FR: Data curation, Formal analysis, Investigation, Methodology, Project administration, Software, Validation, Visualization, Writing – original draft, Writing – review and editing. KB: Investigation, Project administration, Validation, Writing – review and editing. SG: Methodology, Supervision, Validation, Writing – review and editing. NL: Conceptualization, Funding acquisition, Methodology, Project administration, Resources, Supervision, Validation, Writing – review and editing. NJ: Investigation, Project administration, Validation, Writing – review & editing.

## Footnotes

^1^Of note, we forgot to move one patient closer to the target lights after assessing vestibular function at 1.5 m away from the central light. Thus, this patient was seated further away (1.5 m) from the target lights during the first unweighted session but was moved to the correct distance (1 m) for subsequent sessions. Furthermore, another patient performed gaze shifts at 1.5 m throughout all three sessions due to experimenter error. We retained both patients for statistical analysis because we did not expect this to meaningfully affect our primary outcome, as being seated further away only slightly reduces gaze shift amplitude, and head oscillation ratios are normalized by peak velocity.

^2^Note that visual feedback did not play a (major) role in executing gaze shifts for the presented measurements, as the experiment took place in complete darkness.

^3^For instance, next to the symptom modalities (e.g., pain vs. dizziness) that tend to arise from different organ systems, the affected site of pain in all included patients did not always revolve around the body areas involved in performing large gaze movements: 38.5% suffered from pain in the head area, 30.8% from pain in the neck, and 15.4% from pain in the shoulder region.

^4^The present data allow analysis of gaze stability and can be openly accessed under the link provided in the data availability statement of this manuscript.
